# Rethinking the Animal Shelter's Role in Free-Roaming Cat Management

**DOI:** 10.3389/fvets.2022.847081

**Published:** 2022-03-08

**Authors:** Kate F. Hurley, Julie K. Levy

**Affiliations:** ^1^Koret Shelter Medicine Program, School of Veterinary Medicine, University of California – Davis, Davis, CA, United States; ^2^Maddie's Shelter Medicine Program, College of Veterinary Medicine, University of Florida, Gainesville, FL, United States

**Keywords:** feral cats, community cats, Trap-Neuter-Return, lethal management, cat population control, animal shelter, wildlife

## Abstract

Substantial societal investment is made in the management of free-roaming cats by various methods, with goals of such programs commonly including wildlife conservation, public health protection, nuisance abatement, and/or promotion of cat health and welfare. While there has been a degree of controversy over some of the tactics employed, there is widespread agreement that any method must be scientifically based and sufficiently focused, intensive and sustained in order to succeed. The vast majority of free-roaming cat management in communities takes place through local animal shelters. Throughout the 20th century and into the 21st, this consisted primarily of *ad hoc* admission of cats captured by members of the public, with euthanasia being the most common outcome. In North America alone, hundreds of millions of cats have been impounded and euthanized and billions of dollars invested in such programs. Given the reliance on this model to achieve important societal goals, it is surprising that there has been an almost complete lack of published research evaluating its success. Wildlife conservation and public health protection will be better served when debate about the merits and pitfalls of methods such as Trap-Neuter-Return is grounded in the context of realistically achievable alternatives. Where no perfect answer exists, an understanding of the potential strengths and shortcomings of each available strategy will support the greatest possible mitigation of harm—the best, if still imperfect, solution. Animal shelter function will also benefit by discontinuing investment in methods that are ineffective as well as potentially ethically problematic. This will allow the redirection of resources to more promising strategies for management of cats as well as investment in other important animal shelter functions. To this end, this article reviews evidence regarding the potential effectiveness of the three possible shelter-based strategies for free-roaming cat management: the traditional approach of *ad hoc* removal by admission to the shelter; admission to the shelter followed by sterilization and return to the location found; and leaving cats in place with or without referral to mitigation strategies or services provided by other agencies.

## Introduction

Significant investment is made in active management of cats in many parts of the world, with common (and sometimes purportedly conflicting) goals including reduction of cat populations and associated harmful impacts on wildlife; mitigation of nuisance complaints and public health concerns; and promotion of cat health and welfare. This paper will review traditional and emerging strategies to achieve these commonly held goals, with an emphasis on those available to shelter-based control programs which represent one of the most common contexts through which cat management efforts are funded and delivered.

Definitions and distinctions amongst cats have been made based on socialization level toward people (e.g., “feral” or unsocialized vs. friendly), ownership status (e.g., owned/pet vs. semi-owned or un-owned), confinement (indoors, outdoors at times, or free-roaming), level of care (subsidized or self-sufficient), or location found (urban, suburban, or natural habitats). Assessment of the category(s) to which a free-roaming cat belongs often cannot be determined on casual inspection, cats may move between categories over time, and broadly applied interventions will inevitably impact cats in multiple categories. Therefore, this review will focus on the potential for cat management practices to achieve common societal goals with respect to any cat found outside without evidence of ownership, for which the umbrella term “free-roaming cats” will be used.

### Background

In recent decades, there has been extensive research and public debate on the role of lethal and non-lethal methods of free-roaming cat management (1, 2). A point of agreement among advocates of either approach is that cat management strategies should be subject to scientific scrutiny, driven by data, and reviewed for impact with reference to the specific environment in which they are applied and the outcomes they are intended to achieve (1, 3). In this context it is appropriate to evaluate all cat control methods that might be applied on a broad scale by the same standards, including cost, effectiveness, and practicality on a large scale.

#### Trap-Neuter-Return

Trap-Neuter-Return (TNR) in particular has been the subject of extensive scientific and public debate (2, 4, 5). TNR programs have most commonly relied on community volunteers to trap cats for sterilization (often with vaccination for infectious diseases and ear tipping to identify cats as sterilized) and return to the location found. Trapping is usually on an *ad hoc* basis based on volunteer capacity and driven by local concern or annoyance regarding individual cats or larger groups and is often associated with adoption of some cats, especially socialized kittens. Demonstrated benefits of TNR include improvement to cat health and welfare and reduction of nuisance complaints (6–8). Success has also been reported in reducing or eliminating cat populations in focal areas (9, 10). Volunteer engagement means that TNR programs are often carried out at minimal or no public cost (11, 12). However, population models differ on their predictions of what proportion of cats must be sterilized to meaningfully reduce or eliminate cat populations on a broad scale (2, 13–17).

#### Non-shelter-based Alternatives to Trap-Neuter-Return

Alternatives to TNR include lethal and non-lethal cat control methods implemented by federal or local wildlife management programs. Like TNR, some of these interventions have been well-documented and studied, with varying results. Successful campaigns are expensive and labor intensive, with costs to eradicate cats from islands ranging from $400 to $431,000 USD per km^2^ (18). At the lowest end of that range, a campaign to eliminate 40 cats from Faure Island (58 km^2^ area primarily using aerial distribution of poison bait) took just 3 weeks and cost $26,000 (19). At the higher end of the range, eradication of 761 cats from Macquarie Island (128 km^2^ area primarily using cage traps and shooting of cats) took 22 years and cost ~$2.5 M (20). Recently, the proposed eradication of the estimated 1,629 feral cats from Australia's 4,405 km^2^ Kangaroo Island, which is inhabited by ~4,400 people, via a culling campaign including poisoning, trapping, and shooting was projected to cost $15 million over a 10-year period (21). The largest documented primarily non-lethal cat removal campaign took 3 years and $2.9 M to eliminate 66 cats from San Nicolas Island (57 km^2^ area primarily using padded leg-hold traps) (22). Where complete eradication and exclusion of new immigration is not possible, significant ongoing investment is required on top of initial costs (23).

#### Shelter-Based Methods of Free-Roaming Cat Management

The methods and cost of such intensive campaigns preclude their use on a large scale in areas inhabited by people and pets (24). In the absence of large-scale government-sponsored alternatives, this leaves the vast majority of cat management on a community level to take place through programs operated by local animal shelters, including publicly and privately funded organizations. Billions of dollars are invested annually and millions of cats pass through shelters each year (25). In spite of this significant investment and the implied or stated reliance on these programs as the primary alternative to TNR, curiously little scientific scrutiny has been applied to the potential for traditional or emerging sheltering methods to decrease cat number or mitigate the impacts of cats on wildlife or public health.

This is an important oversight. TNR has been criticized because it may fail to reach the necessary scale or be sustained with sufficient intensity to meaningfully reduce cat populations. However, the same can be said of shelter-based control programs. Animal shelters are not generally staffed or funded at the level of documented successful cat control campaigns. Even the lowest cost documented for such campaigns, at $26,000 USD for 40 cats ($650/cat) (19), would be substantially out of reach for community animal control programs.

The methods as well as the cost of meaningful eradication campaigns are a limiting factor for shelter-based control. Use of poison, shooting, or other broadly applied lethal methods is not an option in most populated regions. This leaves community control efforts mainly reliant on live capture (either trapping or confinement by some other means), a process that requires the location and habits of cats to be known with some precision. Traps set by shelter personnel can be interfered with or destroyed unless continually monitored. As a result, the vast majority of shelter-based control involves cats that are brought into shelters on an *ad hoc* basis by individual members of the public, unrelated to targeting of particular cats or locations with respect to wildlife protection.

Thus, traditional shelter-based control shares some of the same potential weaknesses raised by critics of TNR as a means to meaningfully reduce cat populations. However, advocates for traditional shelter methods might argue that cat management programs in populated regions have additional goals. For instance, TNR programs have been touted as a means of improving cat welfare, decreasing disease transmission, and reducing nuisance complaints. These objectives are shared by most shelters, along with a priority placed on reuniting lost animals with their owners and finding new homes for pets in need (24, 26).

The extent to which traditional shelter-based control programs attain commonly held goals will benefit from the same critical examination applied to TNR and other methods. Discontinuing ineffective strategies will allow greater investment in more impactful approaches. At the same time, addressing problems for which animal shelter programs have been a perceived palliative—such as conservation of birds and wildlife—would benefit from a recognition of the true potential and limitations of the shelter-based tactics on which advocates have historically relied. These methods can be broadly divided into three categories: removal (whether for adoption, relocation, or euthanasia); sterilization and return to the location of origin; or leaving cats in place with or without referral to additional resources. In one form or another, these three categories encompass all possible responses to cats in the community.

### Free-Roaming Cat Dynamics and Public Perception

Strategies for cat management in populated areas must account for the number and dynamics of free-roaming cats as well as the nature of public perception and preferences with regard to these animals. Although estimates for cat population size vary widely, the numbers are unarguably substantial: 30–80 million unowned and 70–100 million owned cats in the United States; and 10 million owned and 1.4–4.2 million unowned cats in Canada (25, 27). Between 25–85% of owned cats are kept indoors in the United States and Canada (25, 28) and >80% are sterilized (29), suggesting that while management strategies must account for both groups, unowned cats likely contribute the most to cat population replenishment and account for the majority of concerns. Unowned cats will also generally be found at higher density in modified environments where shelter-based programs tend to predominate, vs. natural habitats in which other methods may be deployed (30).

Importantly, although public and published debate has tended to center on cat “colonies” (cats living in large aggregates around a food source), such groups account for <5% of unowned cats (31–35). Scattered individual cats accessing multiple food sources are difficult to detect compared to the more visible and troublesome groups. Identification and management of dispersed cats in urban and suburban areas relies almost exclusively on the voluntary actions of community members who are in a position to notice one or a few free-roaming cats in their immediate neighborhood and raise concerns or complaints.

Reliance on public participation for management of most cats means that attitudes toward cat control must underpin any successful strategy. Multiple surveys have documented a majority of community support for TNR in the US and Canada (36, 37).

There is less data on support for management of stray cats through shelters detached from the question of euthanasia. A California survey found that 76% of respondents favored spay/neuter and return as a management strategy, while 73% also supported impoundment of stray cats and dogs (31). Although this survey did not distinguish between preferences for impoundment of cats vs. dogs, it suggests that at least under some circumstances a majority of the public are supportive of options for shelter admission as well as TNR for community animal management.

However, where shelter admission is explicitly linked to euthanasia, support falls off substantially. For instance, in a survey in the US, >80% of survey respondents reported that they would leave a cat where it is if the alternative was that the cat would be killed (38). A survey in Guelph, Ontario found that respondents believed that euthanasia at shelters was the least effective method for managing free-roaming cats, ranking only above “do nothing.” Qualitative follow-up uncovered moral discomfort even where euthanasia was considered a theoretically effective option (39). In contrast, accessible spay/neuter, cat owner education, and TNR were deemed effective by more than three-quarters of respondents.

Taken together, these data indicate that many community members simply will not cooperate with shelter programs or access shelter resources if they believe the result will be probable death of a cat. Some people will continue to tolerate nuisance behaviors that could be at least partially mitigated through sterilization of the cats and education of known caregivers. Pet cats may be abandoned or added to colonies if their owners can no longer keep them but fear bringing them to a shelter. In the worst case, cats will continue to breed unchecked, and a handful of cats that might have been manageable will grow into a colony creating significant public health and wildlife risks.

### Harm Reduction Opportunities Aligned With Public Preference

The problem of free-roaming cat management may benefit from the “harm reduction” approach, which has been impactful in the public health sector (40). Harm reduction methods recognize that while elimination of an undesired behavior (such as intravenous drug use or teen sexual activity) may be ideal, it is not always achievable (41). Paradoxically, interventions that acknowledge that the behavior will sometimes occur but that aim to reduce the associated risk (such as clean needle exchange programs for IV drug users or access to birth control for teenagers) have been found to be more effective at reducing negative consequences than strictly abstinence-oriented approaches. Similarly, while there have been calls to eliminate cats on the North American continent “by any means necessary” (5), practical considerations limit the possibility that such an outcome can be achieved. Shelters practicing lethal methods create significant barriers to engagement, education, and risk mitigation, paradoxically increasing the harmful impacts they aimed to prevent. Conversely, non-lethal programs may open up opportunities to significantly mitigate risks to cats and reduce their impact on the environment. Consideration of such opportunities is an important element of evaluating the three possible shelter-based approaches provided here (removal, return, and remaining in place) (2, 12).

## Removal From The Environment As A Shelter-Based Control Strategy

Removal refers to any action that results in a cat being taken from the environment where it was living and not returned. In most non-shelter management contexts this has generally meant that cats were killed, but there have been some cases of removal for relocation (22). From an ecosystem perspective, the impact of removal will be the same regardless of whether cats are killed, relocated or adopted.

Historically, shelter-based management of cats relied almost exclusively on removal, and this remains a common practice. Impounded cats may be reclaimed by their owners, adopted, relocated, or euthanized, but are not returned to the location found.

There has been a tendency to assume that removal to animal shelters is a more effective method for management of free-roaming cats compared to TNR. This likely reflects the intuitive belief that removing a cat leads to the presence of one less cat in the environment, while sterilizing and returning a cat clearly does not reduce the population by one. However, this simplistic view fails to take into account what happens when density is reduced and immigration or breeding by remaining individuals is not prevented.

In order to avoid rapid repopulation, it has been well-documented in many species that removal must reach a critical threshold. For instance, removal of over 50% of coyotes over a 2-year period resulted in drastic initial population reductions (42). However, the population rebounded to pre-removal levels within 8 months as a result of increased litter size and survival. This tendency of populations to rebound to the carrying capacity of the environment may be the basis for the old saying “Kill a coyote and two will come to its funeral.”

When thus placed in the contexts of coyotes (or other highly adaptable species with whom we share urban and suburban environments, such as rabbits or racoons), the limitations of removal may seem obvious. One can easily imagine that if there are 10 coyotes living in a field and 1 or 2 are removed—whether killed or relocated—without any other modification to reduce available food or habitat, the remaining animals will quickly repopulate to the carrying capacity of the area.

Perhaps because of the emotionality of the debate about TNR and long-standing acceptance of shelter-removal in North America, the same scenario does not seem so readily appreciated when it comes to cats. It may also be that because cats are considered a domesticated species, there is a tendency to assume that different biological factors will govern their management. However, not surprisingly given their prolific and adaptable nature, the same population dynamics observed in other litter-bearing mammals have been documented in this species. For instance, when 44% of cats on a semi-isolated peninsula were removed through an intensive month-long trapping effort, the number of cats returned to pre-removal levels within 3 months (43).

On a larger scale, the critical threshold for cat population control through removal has been estimated at 50% or more in multiple modeling studies (13, 14, 16, 17). While lower than the estimates of 57% to >90% for TNR to reach a threshold of control (13, 14, 17, 44, 45), this level is still substantially out of reach for shelter-based removal programs. For instance, even at the low end of the estimated range of unowned cat populations in the US, 50% removal would require admission of 15 million cats to shelters (over 13 million more than the ~1.34 million free-roaming/stray cats estimated to enter shelters in 2019) (46).

The gap between the number of cats currently removed and the number required to reach the critical threshold becomes even more striking when considered on a rolling vs. annual basis. Although shelter intake does show seasonality in association with a rise in summertime kitten births, admission of breeding-age animals is distributed throughout the year vs. intensively as generally modeled or applied in focused control efforts. A total of 1.34 million cats admitted annually averages to 3,659 per day, or 1 in ~8,200 cats at the low end of the estimated unowned cat population range. There is simply no plausible biological basis to support the idea that untargeted removal of fewer than 1 in 8,000 animals on a day to day basis is effective for control.

### Negative Consequences of Failed Removal Efforts

Importantly, removal short of eradication may not only fail to decrease the population, it can magnify the concerns associated with each individual. The increased breeding, birthing, and translocation of animals documented in response to lower population density has the potential to increase disease transmission opportunities and risk. Animals migrating from one location to another may introduce novel pathogens to the resident populations, including zoonotic infections. Young animals are more susceptible to contracting and shedding a number of pathogens of concern for public and/or wildlife health, such as roundworm, hookworm, and toxoplasmosis.

A juvenile-shifted age structure may also have welfare implications for the animals themselves (47). In litter bearing species, young animals suffer substantially higher rates of mortality compared to adults. For instance, the mortality rate for kittens born to free-roaming cats is as high as 75% (48), while the mortality rate for free-roaming adult cats has been estimated to be as low as 10% (49). Thus, although preserving the welfare of cats is an important goal for most shelters, by triggering an increased birth rate in response to lower density, untargeted removal may lead to a paradoxical increase in the number of kittens suffering and dying (16).

In addition to shifting the age structure toward younger animals, greater environmental harm and risk may also result from a paradoxical increase in overall population size in response to removal. For instance, researchers evaluated the impact of removing up to 30% of cats from target areas (50). This study was intentionally designed to replicate what could be realistically achieved in open cat populations vs. the highly localized contexts in which successful eradication has been documented. Contrary to expectation that substantial removal would decrease population size, the number of cats present in the culling sites *increased* by 75% to over 200%. The authors speculated that this resulted from immigration of new individuals in response to removal of the most dominant adults. When culling was discontinued, cat numbers fell and stabilized at pre-culling levels. This led the authors to conclude: “*This study provides evidence that ad hoc culling of feral cats may be not only ineffective, but has the potential to increase the impact of feral cats in open populations*.” This is an important and striking finding. Although in this case the cats were culled, removal to shelters also results in decreased density at the source and similarly takes place in open populations in which new immigration cannot be prevented. This suggests that the practice of *ad hoc* admission to shelters may not only be ineffective, but may actually increase harm to the wild populations it has aimed to protect.

### Opportunity Costs of Failed Removal Efforts

A final risk of untargeted removal is the potential opportunity cost of reliance on an ineffective method to solve genuine problems. In addition to diverting resources, this may reduce incentives to identify and implement more effective solutions. Again, the management of other prolific and adaptable species provides informative parallels. For instance, the harm of reliance on ineffective methods has been hypothesized in the context of managing coyotes through removal/lethal control by federal wildlife personnel:

“*As long as private livestock producers can externalize the costs of predator losses via government-subsidized predator control, they will have little incentive for responsible animal husbandry techniques, i.e., reduce stocking levels, clear carcasses and after-births quickly, confine herds at night or during calving/lambing, install fencing…or numerous other non-lethal preventive methods to avoid depredation*.” (51)

This theoretical scenario played out in Marin County, CA, when lethal management of coyotes was banned in the region. Producers responded by installing electric fencing and reduced livestock losses by 60–70% (52). These interventions were available previously but were not utilized to the fullest extent until the false promise of lethal control was eliminated.

Similarly, there are a number of mitigation strategies for cat-related concerns, from resolving nuisance situations to protecting wildlife and reducing public health risks. Releasing reliance on ineffective removal programs may free up resources to invest in emerging shelter-based programs as well as non-shelter-based solutions that better address these issues.

The limitations and potential harms of untargeted shelter-removal have led to a growing number of recommendations against this practice (26, 53) as reflected in the 2021 position statement from the National Animal Care and Control Association.

“*It is the position of NACA that indiscriminate pick up or admission of healthy, free-roaming cats, regardless of temperament, for any purpose other than TNR/SNR, fails to serve commonly held goals of community animal management and protection programs and, as such, is a misuse of time and public funds and should be avoided*.” (54)

### Appropriate Use of Removal by Shelters

In spite of its limitations for cat management on a large scale, there are appropriate uses for shelter removal (impoundment) of healthy cats in specific circumstances. As with TNR or other methods, *sufficiently targeted and sustained* removal has the potential to decrease or even eliminate focal groups of cats where a critical need is identified, such as in vital habitat or severe nuisance situations (16, 17). Achieving the necessary level of intensity and public support generally requires a multi-faceted approach by which friendly cats are adopted out, healthy but unsocial cats are relocated, and seriously ill or suffering cats are treated or humanely euthanized. Focused and sustained follow-up is required to ensure that populations do not rebound and should be included in the planning for any intervention effort targeting a group of cats.

Impoundment of individual cats may also be indicated under specific circumstances. This would generally include sick and injured cats and those at exigent risk due to immediate environmental factors (e.g., trapped on a median strip of a busy road, living in a building scheduled to be demolished). Impoundment would also be an appropriate response for a cat known to have been abandoned, for instance when a neighbor is aware that an owner has moved away and intentionally left the cat behind. It may also be appropriate for cats that are not sick or injured but are not thriving where they are (e.g., poor body condition, matted fur). In addition, where possible kittens should be adopted into pet homes whether through a shelter or by community volunteers.

## Sterilization and Return As A Shelter-Based Control Strategy

In recent years, an increasing number of shelters have added shelter-based sterilization and return to their methods of free-roaming cat management. This has emerged as the first wide-scale alternative to shelter-based removal programs that predominated in North American shelters for more than a century. Sometimes referred to as Shelter-Neuter-Return (SNR), Return-to-Field (RTF), Return-to-Home (RTH) or the umbrella term “Community Cat Programs” (CCP), this approach is similar to traditional TNR in that cats in good health are sterilized, vaccinated against infectious diseases, ear-tipped, and returned to the location found (either by the finder, shelter staff, or volunteers). However, it should be noted that shelters may provide veterinary care and other services in support of community/volunteer TNR programs.

The primary differentiation of shelter-based return from TNR is the origin of the cats. In TNR programs, cats are typically trapped and transported to veterinary providers by community cat caregivers or volunteers associated with community TNR groups with the specific intent of returning the cats to their original location following sterilization. In shelter-based return programs, cats are trapped and transported to the shelter by individuals or animal control officers seeking help from the shelter for welfare, nuisance, or environmental concerns. Thus, shelter-based return has the potential to reach a wider swath of free-roaming cats than those that come to the attention of advocates in the community, notably the majority of free-roaming cats that live outside of highly visible groups clustered around a food source.

Although originally conceived as an alternative to euthanasia for cats that could not be adopted from shelters, additional benefits of shelter-based return programs have come to light, leading to their expansion to include friendly as well as feral free-roaming healthy cats. Importantly, when returned to the location of origin, cats in good body condition are likely to continue accessing whatever food source was available previously, rendering that food unavailable to other intact cats in the area and preventing the increased breeding and immigration that occurs in response to removal.

A reduction in kitten births and/or decreased translocation of individuals could explain the decrease of 29–38% in cat and kitten intake and 20–29% decrease in the number of cats picked up dead on the road reported in conjunction with shelter-based return programs (55, 56). While many factors can lead to changes in shelter intake to shelters, the decrease in these communities contrasted with steady or increasing cat intake in the years prior to the program in spite of removal by impoundment of thousands of cats annually.

### Harm Reduction Opportunities in Conjunction With Shelter-Based Sterilization and Return

Sterilizing and returning cats offers an avenue for engagement with the majority of residents who believe shelters should play a role in stray animal management but are opposed to euthanasia. By engaging this substantial sector of the community, harm reduction opportunities are created that may not have been accessible to either traditional shelter-based removal or traditional TNR programs.

The presence of a cat in good body condition is a de facto indicator that a food source is present in the area where the cat was found. Even where no visible group of cats is present, it is likely that other cats are also accessing a freely available food source. Shelter-based removal generally affords no opportunity or motivation to identify this source, locate other intact cats in the area, educate caregivers, or take other mitigating actions. Shelter-based return, by contrast, affords multiple such opportunities.

Even when the motivation of the cat trapper is resolution of a nuisance issue vs. concern for cats or the environment, these individuals can be an important part of developing long-term solutions. Previously, shelters recognized many trappers who were “frequent flyers,” repeatedly bringing in cats over a period of months or years. Trapping a cat provided short-term relief, leaving little incentive to address the root cause. Predictably, however, another cat would show up soon after one was removed. This is the basis for the common recommendation against trapping and relocating nuisance wildlife such as racoons or squirrels: without addressing the instigating conditions, more will eventually appear.

By contrast, when trappers are advised that cats will be returned, they can be engaged in identifying longer-term solutions. In some cases, this will involve the trapper themselves modifying their environment in some way, such as placing a lid on an uncovered trash can or bringing a bowl of dog food in from the porch. This has the benefit of reducing environmental carrying capacity (and also reducing food sources for other potentially problematic wildlife species) as well as helping to resolve complaints.

There is also a direct benefit of sterilization in reducing roaming and nuisance behaviors along with improving cat health (57). Trappers are familiar with their own neighborhood and can often identify one or more additional cats that can be targeted for follow-up TNR. In this way, engagement with complainants can help to micro-target sterilization efforts and identify those cats living outside of highly visible groups.

Return of cats can also lead directly to opportunities for education of caregivers and further mitigation. At the time of return, the cat itself may lead shelter staff to a food source and additional cats, including cats unknown to the original trapper that may be an ongoing source of kittens. Additionally, many such programs include hyper-local outreach coordinated with cat return, e.g., by placing flyers in multiple languages on nearby doors or even placing breakaway collars on the cats with information about the program. Offering low-cost spay/neuter for additional cats in the neighborhood can open the door to education on responsible feeding practices, management of cat waste and other mitigation strategies.

The practical value of engaging with caregivers and concerned community members should not be underestimated. Feeding of cats is a common behavior: between 7% and 26% of survey respondents in various studies in the US reported feeding cats they do not own, with the average number of cats fed ranging from 2.6 to 4 (31–34). Feeding of just a few cats is virtually impossible to regulate as it can take place quickly, anonymously, and on private property. Yet providing excessive food will tend to increase breeding and immigration as surely as will reducing cat density. This can be mitigated by guiding caregivers to feed only as much as the cat(s) will eat in 30 min or less; feeding on an elevated surface to limit access by other animals; keeping the feeding area clean and in one spot; and feeding at a consistent time of day to enable identification of which cats are present, note any newcomers, and take needed action (e.g., trap and sterilize).

## Leaving Cats in Place With or Without Referral to Additional Resources

**Figure 1:**
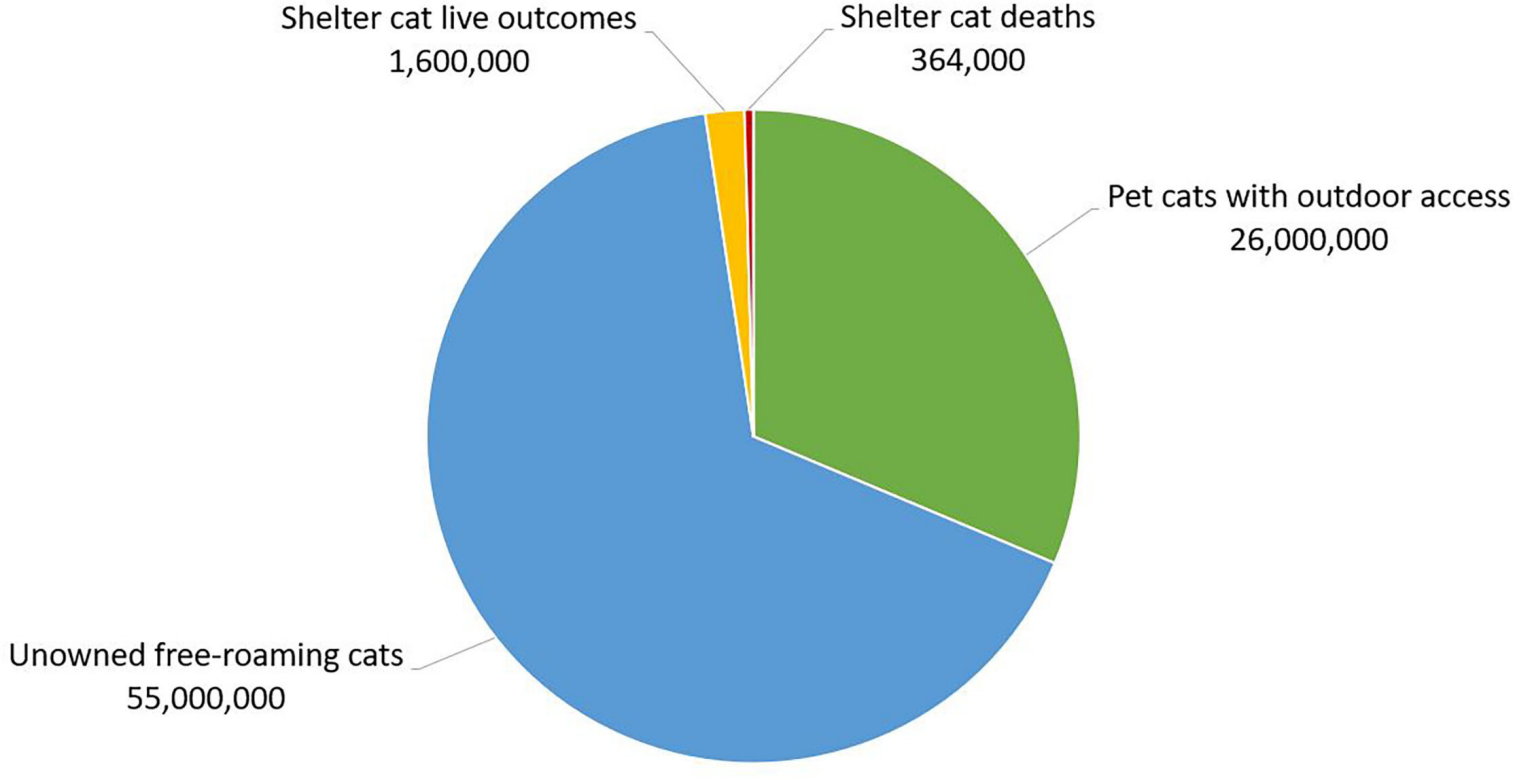
Estimate of the number of cats in the United States with outdoor access and the number and outcome of cats taken in by animal shelters. Approximately 33% of an estimated 79 million owned pet cats are allowed outdoors at least some of the time (58). The number of unowned free-roaming cats has been estimated at 30-80 million, so a mid-point of 55 million was used (19). In this example, almost one-third of outdoor cats are owned pets, two-thirds are un-owned free-roaming cats, and <3% are managed by animal shelters (12).

Given the reality that fewer than 1 in 8,000 free-roaming cats are admitted to North American shelters on a daily basis, remaining in place is the status quo for the vast majority as shown in Figure 1. Additionally, for some shelters, sterilization-return is not an option due to lack of veterinary services, financial limitations, or legal barriers. For these shelters, leaving in place is the only alternative to removal. Even for shelters with ample resources, there may be instances where shelter admission is not the most effective or humane way to address a concerning situation. There is an increasing recognition that community-based care and services are often more equitable and humane as well as preferable to the costs and risks associated with shelter impoundment (59).

With these considerations in mind, the option of leaving cats where they are may benefit from more intentional use as a management strategy rather than simply being the default option most of the time. By recognizing that most cats will remain in the community, shelters can be more strategic about which cats are admitted while also investing resources in stabilizing populations and reducing harms associated with cats who remain outside the shelter's walls.

### Harm Reduction Opportunities in Conjunction With Leaving Cats in Place

Leaving cats in place need not be a passive practice. Although shelter-based sterilization and return is a powerful means to open doors for communication, even a call about a cat can become an opportunity for engagement. As with other species with which we share urban or suburban environments, support and education can be provided to mitigate nuisances and reduce risks associated with cats. Strategies for coexistence include reducing attractants such as food sources, using chemical or motion activated repellants, and modifying habitat to exclude or discourage entry. These strategies are commonly recommended in the context of urban wildlife not out of any particular advocacy for racoons, skunks, squirrels or other species sometimes looked upon as pests, but rather out of simple recognition of the futility or potential harm of removal or relocation.

In the case of cats, coexistence strategies can be combined with education of cat caregivers to feed appropriately, manage waste, and most importantly, to access available services to get cats sterilized. Even where the shelter is not able to offer sterilization services directly, they may be able to provide vouchers, loan traps or even assist with transport to a local clinic.

### Additional Considerations for Leaving Cats in Place/Diversion Directly to Sterilization Services

#### Shelter Operations Impact

Shelters have a number of critical roles to play in communities, including admitting and caring for sick and injured animals, protecting animal victims of cruelty and neglect, and rehoming pets whose owners can no longer care for them. In many communities, shelters are also on the front lines of response to disasters and other emergencies, supporting pet owners by providing safe harbor for animals in danger or distress. Protection of public health and safety is another essential shelter function, including response to dangerous animals and mitigation of zoonotic disease threats. In addition to these important reactive functions, shelters ideally serve communities best when they are able to support community members and prevent problems from developing in the first place.

When shelter resources are not overstretched by unregulated intake of healthy free-roaming cats and resultant crowding within the facility, they are better able to perform these critical functions. This was seen in many regions as intake dropped dramatically during the first year of the COVID-19 pandemic (60). Shelters that previously may have euthanized even mildly ill or injured animals found themselves with the resources to care for these most vulnerable pets. They were also able to provide safe temporary housing for animals in need, whether due to natural disaster, because the owner was sick with COVID-19, or another exigent need.

#### Effect on Adoptions and Rehoming Success

When shelters are not overcrowded, they are better able to provide a safety net for owned pets, reducing the number abandoned or relinquished to shelters. Even when animals do need to be admitted, the chances for adoption will be greatly increased with less crowding and competition. Fewer cats in the building means staff and volunteers can provide a higher level of care, enrichment, and treatment for each individual, reducing the number of pets that end up euthanized. This in turn is likely to improve public confidence and further reduce new abandonment of cats to the outdoors by owners who otherwise would have been reluctant to entrust their pet to a shelter's care.

#### Lost Pet Reunification

Reuniting lost pets with their owners is a central goal of most shelters. Contrary to the historic assumption that this goal was well-served by bringing cats to a shelter facility, leaving healthy cats in place (or returning them to the location found) may be a far better means to achieve this end. Multiple studies have now documented that cats are 10–50 times more likely to be reunited with their owners by returning home on their own or being found in the neighborhood of origin than through a call or visit to a shelter (61–63). This reality is reflected in the fact that only ~ 2% of cats admitted to US shelters are reunited with their owners (64).

The low rate of owner reunification for shelter cats likely reflects common behavior patterns of both cats and owners. Allowing cats outdoor access is still a common practice in many communities, and a search for a missing cat may not be initiated until well past even the longest stray holding period in a shelter would have expired. At the same time, lost cat behavior differs from dogs. Often a “lost” cat is missing because it got trapped somewhere, was frightened and went into hiding, or perhaps most commonly, was simply enjoying a meal at another neighbor's house when a well-intended “Good Samaritan” intervened and brought the cat to a shelter. Thus, a cat may not appear in a shelter until days or even weeks after it went missing, again resulting in a mismatch between the timing of when cats are lost, when owners look for them, and when cats are in shelters.

Impoundment of free-roaming cats may disproportionately impact lower-income families, as barriers of transportation, language, cost, or simple lack of awareness of the cultural practice of impounding cats may deter pet owners from seeking their lost cat at a shelter. This may account, at least in part, for the fact that people earning < $30,000 per year were only 1/10th as likely to find a lost cat as those earning >$50,000 (63).

A more equitable, as well as more effective, approach may be to help finders reunite most lost cats with their owners without impoundment at a shelter. This could include posting photos on the shelter's lost and found website, offering services to scan found cats for microchips, and encouraging finders to post on local social media, talk to neighbors, post signs locally, and even consider placing a paper “is this your cat” collar on the cat. Advising finders not to feed cats that show up in their yards in good body condition may also encourage cats to simply go back home. Exceptions to this policy should be made whenever cats are sick, injured, in poor body condition or otherwise failing to thrive, or after efforts to reunite the cat in the neighborhood of origin have failed to identify an owner or caretaker.

#### Ecosystem Impact

Perhaps the most sweeping, though counterintuitive, benefit of leaving cats in place is simply the inverse of removal. As described above, untargeted removal of cats or other litter-bearing mammals leads to a destabilization of age and dominance structures, resulting in a paradoxical increase in numbers as well as potential harms. Impounding, caring for, and potentially euthanizing healthy free-roaming cats also diverts resources which could be better invested proactively.

By replacing *ad hoc* admission with solutions to sustainably reduce free-roaming cat populations to the extent possible, leveraging spay/neuter, and minimizing additional costs of impoundment by diverting most healthy adult free-roaming cats to community-based services, the overall number as well as the harmful impact and risks experienced by individual cats can be more effectively reduced.

## Selecting The Appropriate Shelter-Based Strategy

Each of the three strategies available to community animal shelters for free-roaming cat management—removal, sterilization and return, or leaving cats in place—is appropriately used under certain conditions. In non-emergency situations (e.g., the cat is not sick, injured, causing or experiencing immediate danger), an assessment should be performed of the individual cats' circumstances and the environment in which it was found. Most healthy free-roaming cats should be referred to community resources or admitted for sterilization and return. Exceptions should be made where evidence exists of abandonment or other change in circumstances resulting in increased risk, such as where an owner is known to have left the area recently. Cats should also generally be removed rather than returned where a significant concern exists, such as presence within a critical habitat for prey species. For example, in Alachua County Florida, animal shelter and conservation managers collaborated to develop a policy to protect both wildlife and cats. The policy supports a relocation program for cats on properties specifically managed for conservation and a plan for collaborative assessment and mitigation of conservation threats on properties that are not formally defined as conservation areas. If removal is pursued in the latter case, it should be coupled with resources to meaningfully abate the issue including sufficiently intensive and sustained removal efforts and prevention of new immigration or abandonment.

In order to perform the necessary individualized assessment to tailor an appropriate response, an increasing number of shelters are replacing *ad hoc* admission with a more thoughtful approach, sometimes termed “Managed Admissions” or “Coordinated Care” (65–69). In this individualized case management approach, contact via phone or web form is made before the animal is transported to the shelter, and a situational assessment is performed to determine the most appropriate course of action. Similar to calling an “advice nurse” prior to scheduling a doctor visit, this provides an opportunity to gather information, identify whether shelter admission is the best solution, and provide alternatives where indicated.

## Conclusion

No realistically available intervention is sufficient to completely eliminate free-roaming cats from the landscape. Traditional *ad hoc* admission to shelters is not a panacea that eliminates the concerns generated by free-roaming cats. Placing it as such in contrast to TNR has needlessly pitted the interests of cats, cat lovers, and shelter staff against the interests of wildlife advocates and public health officials. Success in solving the complex issues associated with free-roaming cats will be best served by moving beyond this false dichotomy to an evidence-based assessment of all possible approaches to management of this prolific and adaptable species.

In many communities, animal shelters will continue to play a central role in response to free-roaming cats and the concerns they generate. This role will be carried out most effectively when all sheltering agencies, public and private, are able to tailor their responses to the needs of each situation encountered: for each individual cat, is it better to be admitted to a shelter, to be altered and returned, or to remain in place with referral to resources for coexistence? We owe it to ourselves to ask this question without prejudice or pre-conceived notions, for each and every cat that might come through a shelter's doors. The result will be solutions that balance the needs of wildlife, public health, pets, and community members to the greatest possible extent and make the most effective use of all available resources.

## Author Contributions

All authors listed have made a substantial, direct, and intellectual contribution to the work and approved it for publication.

## Conflict of Interest

The authors declare that the research was conducted in the absence of any commercial or financial relationships that could be construed as a potential conflict of interest.

## Publisher's Note

All claims expressed in this article are solely those of the authors and do not necessarily represent those of their affiliated organizations, or those of the publisher, the editors and the reviewers. Any product that may be evaluated in this article, or claim that may be made by its manufacturer, is not guaranteed or endorsed by the publisher.
